# Apalutamide‐Induced Hypothyroidism Associated With Increased Thyroid Hormone Clearance in Metastatic Prostate Cancer Patient: A Case Report

**DOI:** 10.1002/iju5.70161

**Published:** 2026-03-08

**Authors:** Jun Furumido, Akihiro Yamashita, Ryo Kato, Kanta Hori

**Affiliations:** ^1^ Department of Urology Asahikawa Kosei General Hospital Hokkaido Japan

**Keywords:** apalutamide, hypothyroidism, prostate cancer

## Abstract

**Introduction:**

Apalutamide (APA) is an androgen receptor signaling inhibitor widely used for metastatic hormone‐sensitive prostate cancer (mHSPC), though it can induce hypothyroidism. We report a severe case of APA‐induced hypothyroidism in a patient with a history of total thyroidectomy.

**Case Presentation:**

A 65‐year‐old man receiving levothyroxine (175 μg/day) after thyroidectomy was diagnosed with mHSPC (Gleason score 4 + 4; PSA 13 904 ng/mL) with findings suggestive of cancer‐associated disseminated intravascular coagulation. Following APA initiation (240 mg/day), PSA decreased rapidly to 0.01 ng/mL. However, TSH levels rose progressively despite increasing levothyroxine to 275 μg/day. After a 4‐month follow‐up interruption, TSH reached 187.9 mIU/L. APA withdrawal led to rapid TSH improvement.

**Conclusion:**

Careful TSH monitoring is essential during APA treatment, especially in patients with pre‐existing thyroid dysfunction or prior thyroidectomy. Appropriate levothyroxine titration and multidisciplinary collaboration are essential for the continuation of oncological therapy with APA.

## Keynote Message

We report a case of hypothyroidism, a rare adverse effect of apalutamide. In patients with pre‐existing thyroid disease, apalutamide treatment has been associated with increased thyroid hormone clearance, resulting in decreased serum thyroxine levels and elevated TSH. These findings underscore the importance of careful endocrine monitoring, such as during apalutamide treatment.

**Abbreviations**
ADT: androgen deprivation therapy
APA: apalutamide
ARSI: androgen receptor signaling inhibitor
CRPC: castration resistance prostate cancer
mHSPC: metastatic hormone‐sensitive prostate cancer
PET: positron emission tomography
UGT: UDP glucuronosyltransferase

## 1 Introduction

Prostate cancer is the most frequently diagnosed malignancy among men in many countries and a major cause of cancer‐related mortality [1]. Androgen deprivation therapy (ADT) has long been the mainstay of systemic treatment for advanced prostate cancer. Over the past decade, the introduction of androgen receptor signaling inhibitor (ARSI) has represented a major advance in hormonal therapy for this disease [2, 3, 4, 5]. Among the available medications, apalutamide (APA) was granted FDA approval in February 2018, and in Japan, it received insurance coverage in March 2019 for nonmetastatic castration resistance prostate cancer (CRPC) and in May 2020 for metastatic hormone‐sensitive prostate cancer (mHSPC). APA is a competitive inhibitor of the androgen receptor that suppresses androgen‐mediated transcriptional activity, which is essential for prostate cancer progression. Due to its demonstrated efficacy and manageable safety profile, APA has become widely used in clinical practice. Here, we report a case of APA‐induced hypothyroidism in a patient with metastatic prostate cancer who was receiving levothyroxine sodium hydrate replacement therapy following total thyroidectomy.

## 2 Case Report

**FIGURE 1:**
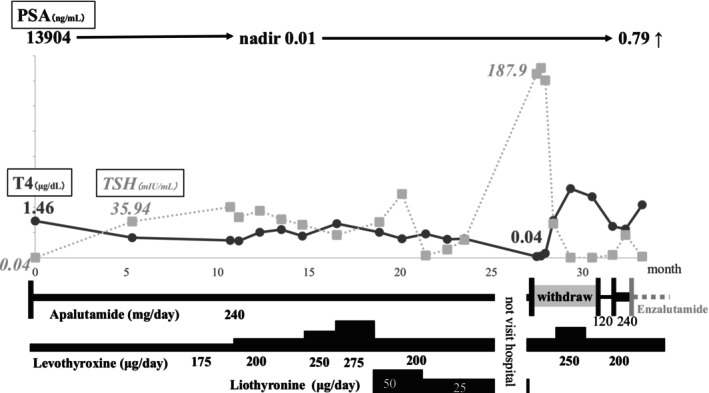
PSA level decreased rapidly after starting APA. TSH level increased to 35.94 mIU/L at 5 months, 50.12 mIU/L at 9 months, and 187.9 mIU/L at 26 months after APA treatment initiation. APA‐induced hypothyroidism was suspected, and TSH level improved rapidly after withdrawal of APA. However, systemic treatment was switched to enzalutamide due to disease progression.

A 65‐year‐old male patient had undergone total thyroidectomy for papillary thyroid cancer 8 years previously and had been receiving levothyroxine replacement therapy. During routine blood testing at an otolaryngology department, markedly elevated coagulation markers were detected: FDP level of 264.1 μg/dL and a D‐dimmer level of 113.0 μg/dL. The patient was subsequently referred to the department of hematology for bone marrow biopsy, which revealed AMACR‐positive adenocarcinoma. Based on these findings, bone metastasis from prostate cancer was suspected and he was referred to our department. PSA levels were elevated at 13 904 ng/mL; however, digital rectal examination revealed no obvious malignant findings. Positron emission tomography (PET) showed heterogeneous uptake in the ribs, right scapula, and spine. Due to right shoulder pain, the patient underwent palliative radiotherapy (30 Gy/10 fr), followed by surgical castration and transrectal prostate biopsy. The diagnosis revealed adenocarcinoma, with a Gleason score of 4 + 4, and the clinical stage was cT1cN0M1 (bone: ribs, right scapula, and spine). He was started on a dosage of 240 mg/day of APA, resulting in a rapid decline in PSA levels to 0.01 ng/mL within 7 months. However, 5 months after starting APA, the TSH level was 35.94 mIU/L, and 17 months later, it remained high, the levothyroxine dosage was increased to 275 g/day. In addition, 50 μg of Liothyronine was used, but thyroid function did not stabilize. After that, he was not allowed to visit the hospital for 4 months. Twenty‐six months later, the TSH level had reached 187.9 mIU/L prompting the suspicion of APA‐induced hypothyroidism (Grade 2). APA was discontinued, after which TSH levels improved, therefore APA was restarted at a reduced dose of 120 mg/day, however, PSA levels were elevated. The dose of APA was increased to 240 mg/day again; regardless of this, the PSA increase was not suppressed, and the treatment was switched to enzalutamide. The clinical course of this patient is summarized in Figure 1.

## 3 Discussion

APA, a novel ARSI, was approved in Japan in March 2019 and has since been widely used in clinical practice. The most commonly reported adverse events are fatigue and skin rash [4, 5]. However, hypothyroidism has also been reported. In the SPARTAN study of nonmetastatic CRPC (nmCRPC), hypothyroidism was reported in 8.1% of patients [4], and in the TITAN study of mHSPC, it was reported in 6.5%–7.1% of patients [5, 6]; but no cases of grade 3 or higher hypothyroidism were reported.

APA induces UDP‐glucuronosyltransferase (UGT) in the liver, promoting the glucuronidation of levothyroxine and subsequent excretion of thyroid hormones in the bile [7]. In other words, the pharmacokinetic interaction between APA and levothyroxine results in an increase in levothyroxine clearance, thereby reducing its therapeutic effect. This results in a reduction of thyroid hormone levels within the body, consequently leading to an elevated TSH level through negative feedback. Additionally, Quattrochi et al. indicate that APA reduces the intestinal absorption of levothyroxine, thereby explaining the higher prevalence of hypothyroidism observed in patients receiving levothyroxine therapy during APA treatment [8]. In the present case, the complete absence of thyroid reserve capacity due to total thyroidectomy when APA‐induced UGT metabolism was triggered led to a significant increase in TSH.

Daviduck et al. reported that patients with pre‐existing hypothyroidism are more susceptible to the thyroid dysfunction during APA therapy, and worsening hypothyroidism was observed in 30% of these patients [9]. Moffatt et al. reported a two‐ to threefold increase in levothyroxine supplementation but a standardized management strategy was not proposed [10]. David et al. summarized three similar cases, all of whom showed rapid TSH elevation 1–2 months after APA initiation [11]. In all three patients, a markedly increased serum TSH level (> 30 mIU/L) was observed within this short timeframe, which was much earlier than the median time to the first TSH elevation of 113 days reported in the SPARTAN study [4]. In some cases, the levothyroxine dose could be reduced after discontinuing APA, while in other cases, normalization required a longer duration. Although there were no data available immediately after the start of APA in this case, the patient's TSH level was as high as 35.94 mIU/L 5 months after starting APA, suggesting that hypothyroidism had already developed at that time and possibly earlier. This clinical course closely resembles that reported in the three previously described cases (Table 1). Furthermore, this case suggests that the patient's physical condition was poor before the sudden rise in TSH levels at 26 months and that he may not have been able to take enough levothyroxine. This finding means the critical importance of close and continuous monitoring in patients receiving levothyroxine therapy.

**TABLE 1:** Comparison of apalutamide‐induced hypothyroidism in patients with pre‐existing thyroid conditions.

Case	Age	Prostate cancer	Patient history	After start APA (month)	TSH (mIU/L)	Levothyroxine increase (%)	Outcome
1	76	nmCRPC	Thyroidectomy	2	81.7	146	APA stopped and returned to normal
2	56	mHSPC	Total thyroidectomy	1	37.0	82	Maintenance
3	64	mHSPC	Autoimmune hypothyroidism	2	35.5	86	Maintenance
This case	60	mHSPC	Total thyroidectomy	5	35.94	157	APA stopped and returned to normal

The standardization of thyroid function monitoring during APA treatment is desirable in future clinical practice. The current guideline recommends measuring TSH and FT4 at baseline, reassessing after 4–6 weeks, continuing at 4–6 month intervals until stabilization, and subsequently performing annual evaluations [12]. However, in this case, TSH and T4 levels underwent a precipitous change following a four‐month period of nonvisitation at the hospital. Particularly in patients with pre‐existing hypothyroidism who are receiving levothyroxine replacement therapy, routine follow‐up may prove to be inadequate in such cases. Consequently, the necessity of monthly blood tests may be necessary. Nevertheless, with careful endocrine monitoring, it is probable that APA therapy can be safely continued.

APA is an effective prostate cancer treatment, but the potential development of hypothyroidism should be closely monitored in patients with pre‐existing thyroid disease. With regular TSH and FT4 monitoring, appropriate levothyroxine supplementation, and collaboration with other departments, we consider that APA treatment can be continued safely, especially in patients with pre‐existing thyroid disease.

## Consent

Informed consent was obtained from all individual participants included in the study.

## Conflicts of Interest

The authors declare no conflicts of interest.

## Data Availability

The data that support the findings of this study are available on request from the corresponding author. The data are not publicly available due to privacy or ethical restrictions.
